# The "impact factor" revisited

**DOI:** 10.1186/1742-5581-2-7

**Published:** 2005-12-05

**Authors:** Peng Dong, Marie Loh, Adrian Mondry

**Affiliations:** 1 Medical Statistics and Epidemiology Group, Bioinformatics Institute, BMRC, A*STAR, Singapore

## Abstract

The number of scientific journals has become so large that individuals, institutions and institutional libraries cannot completely store their physical content. In order to prioritize the choice of quality information sources, librarians and scientists are in need of reliable decision aids. The "impact factor" (IF) is the most commonly used assessment aid for deciding which journals should receive a scholarly submission or attention from research readership. It is also an often misunderstood tool. This narrative review explains how the IF is calculated, how bias is introduced into the calculation, which questions the IF can or cannot answer, and how different professional groups can benefit from IF use.

## Background

The number of periodical peer-reviewed scientific publications is conservatively estimated to exceed 16,000 worldwide; nearly 1.4 million articles are published every year [1,2]. Even though electronic formats theoretically allow access to most current publications, the sum of subscription fees charged by most periodicals exceeds the means of academic institutions, not to mention individuals. Accordingly, librarians must limit the quantity of periodical subscriptions. Researchers have a vast number of journals to choose from when considering where to find information, and where to publish their work. Potential employers of scientists who try to evaluate a candidate's bibliography are aware that not all publications are of equal quality. All three parties need objective, preferably quantitative, information to assist publication and subscription decisions, in effect which publications to count as important. A simple descriptive quantitative measurement of a journal's performance is the "impact factor" (IF), the average number of times articles from the journal published in the past two years have been cited in the current year.

Eugene Garfield, the founder of the Institute for Scientific Information (ISI), proposed a bibliographic system for scientific literature – "Citation Indexes for Science" in 1955 [3]. ISI's database was initially developed for cross reference literature searches and identification of individual scientists working on particular topics [4]. The citation index compiled information that was far more useful and convenient than the usual subject indexing and helped to span the gap between authors and researchers. It mainly consisted of a complete alphabetic listing of all periodicals covered and their representative codes. These codes described the bibliographic category (e.g. editorial, original research, review), while a different set of data was assigned to articles referring to an article in question.

The IF was originally conceived as a quantitative assessment of referenced publications in a given journal found in the scientific literature. By processing the data from the citation index, it became possible to calculate a ratio of cites to a journal. Garfield himself explained the meaning of impact, pointing out that a citation indicates an article has influenced someone and therefore, the more often an article is cited, the greater its influence on the scientific community [5]. This ratio was then used to select the journals for inclusion in the Science Citation Index (SCI) [6]. The journal IF is currently calculated by Thomson ISI for all journals contained in the SCI database, then reported in the Journal Citation Reports (JCR) database. Currently 3700 "world-leading" scientific journals are included in the SCI database [7]. Even more journals are "tracked", which means they are monitored but have not been assigned an IF yet. Inclusion criteria into the SCI database and for assignment of an IF were described in detail by Garfield [8].

It is generally understood that the higher the IF, the "better" the journal. As a result, journals with high IF are often preferentially acquired in institutions where subscription funds are limited; researchers are keen to submit their work to journals with high IF to further their career; the editors of journals with high IF are swamped with manuscripts by researchers who want to publish only with the best; some funding agencies expect their scientists to publish in journals above a certain IF; and recruitment officers tend to look for candidates with publications in high-IF journals.

Unfortunately, the IF alone cannot provide the knowledge needed for informed decision-making. Even more unfortunately so, it is often used simply because it is readily available, while alternative measurements are unknown or unavailable to many decision makers [4].

The present narrative review is an introduction to a field of study within scientometrics, and a basis on which librarians, researchers and funding agencies can discuss the usefulness of the IF in planning their publication and funding strategies. Topics covered include how the IF is calculated, some sources of bias in the calculation, an introduction to some alternative assessment scores that complement the IF, and questions the IF can or cannot answer in the informed decision making process.

## Calculation of the impact factor

The journal IF defined by the ISI is a ratio of two elements. The denominator is the total number of "citable" articles published in a particular journal within a given timeframe. The numerator is the total number of citations in the current year to any article published in this journal during that given timeframe. The ISI has defined this time frame as two years. The IF of a journal A in a particular year *Y *is computed following the formula:



By ISI definition, only research articles, technical notes and reviews are "citable" items. Editorials, letters, news items, and meeting abstracts are "non-citable items" for the purpose of calculating the denominator. All items, however, may be counted in the numerator during the calculation

For example, the *New England Journal of Medicine *published 366 "citable" articles in 2003 and 378 "citable" articles in 2002. Citations in 2004 to any articles published in 2003 and 2002 are 14147 and 14549, respectively. Following the above formula, the IF for this journal in 2004 is:



## Factors that bias the calculation of the impact factor

The ready accessibility of the IF and the lack of other well-known quality indicators have rapidly contributed to the attribution of IF as an indicator of journal quality. However, it is important to remember that the calculation of the IF is biased by many factors. These include:

• Coverage and language preference of the SCI database

• Procedures used to collect citations at the ISI

• Algorithm used to calculate the IF

• Citation distribution of journals

• Online availability of publications

• Citations to invalid articles

• Negative citations

• Preference of journal publishers for articles of a certain type

• Publication lag

• Citing behavior across subjects

• Possibility of exertion of influence from journal editors.

### Journal coverage by ISI

The coverage of journals and the language preference in the SCI database are important contributors to the limitation of the IF. The SCI covers less than one fourth of peer-reviewed journals worldwide, and exhibits a preference for English language journals [9]. Non-English journals have relatively low IFs due to the limited coverage of such journals by the SCI database. Calculation of the IF for non-English journals in their native countries or regions may be a useful way to complement the data in the SCI database [8,10,11]. At the same time, it must be remembered that at present, English is the *lingua franca *of science, just as German was in the 19^th ^and early 20^th ^centuries, and Latin and Greek before that [11]. Further bias has been created by a tendency towards self-citation among American scientists [12].

### Differences across research fields and subject areas

Different citing behavior across subject field imposes a bias on the IF. Articles in rapidly growing areas tend to cite much more recent references than more traditional research fields, in particular theoretical and mathematical areas [13]. This diversity leads to the wide variance of IFs across subject categories. The IF of underrepresented fields is affected negatively [13].

Collecting citations over only two years post publication has an important effect on the IF. Journals in rapidly growing research fields, such as systems biology and bioinformatics, tend to publish papers with a short time interval from submission to acceptance. A large percentage of papers are cited within two years of their publication. This, in result, leads to a high IF. However, there are many journals with longer citation half-lives. Many papers from such journals are still cited frequently much longer than two years after their publication. ISI defines "citation half-life" as the median age of the articles that were cited in the year for which the half-life is reported. Fields with more "durable" literature have a small percentage of short term citations and thus lower journal IF [13]. This field property together with the low number of references per article gives mathematics, for example, a recorded average citation impact that is only a quarter that of biochemistry [14]. Whitehouse [15] has analyzed this for the *British Journal of Radiology *as one example of a journal with long citation half-life. Only 12% of the cites to this journal in 1999 quote the previous two years' publications, but more than 50% of the cites in 1999 to the *BJR *quote papers published in the previous nine years. The scientific impact of the *BJR *is thus underestimated if the calculation is based only on cites to the previous two years' publications. While this affects most journals to some extent, it seems that the highest ranking journals remain quite stable, regardless of the timeframe used for the calculation of the IF [8,16].

A given research field is often also cited by related fields [13]. For example, clinical medicine draws heavily on basic science. As a result, basic research in medicine is cited three to five times more than clinical medicine. The IF is affected accordingly [17,18].

### Differences between journals that have nothing to do with journal quality

A distinct weakness of the IF's algorithm lies in the inclusion of articles into the numerator count that are considered as "non-citable" in the denominator count. Citations to "non-citable" items may dramatically increase a journal's IF [19,20]. Journals publishing large proportion of "non-citable items" can thus achieve higher IFs than journals that predominantly publish "citable" items.

Similarly, the ISI algorithm does not take into account a journals' respective composition of research articles, technical notes and reviews [20]. Reviews are more likely to be cited than original research papers [13,21]. Journals publishing a high proportion of review papers consequently attract more citations and thus are likely to achieve a higher IF.

Editorial preference for longer articles seems to increase a journal's IF. Seglen [21] has shown that the citation rate is proportional to the article length, i.e. longer articles are cited more often.

Given the rapid growth of electronic publications, the online availability of articles has recently become an important factor to influence the IF. Murali et al. [22] determined how the IF of medical journals is affected by their online availability. In that study, a document set obtained from MEDLINE was classified into three groups, namely *FUTON *(full text on the Net), abstracts only and *NAA *(no abstract available). Online availability clearly increased the IF. In the FUTON subcategory, there was an IF gradient favoring journals with freely available articles. This is exemplified by the success of several "open access" journals published by BioMed Central (BMC) and the Public Library of Science (PLoS). Open access journals publish full-text online papers free of subscription fees [23]. BioMed Central (BMC) is an "open access" publisher in business since 2000. BMC hosts over 100 biomedical journals ranging from general interest to specialized research. More than twenty journals published by BMC are currently tracked by the ISI and over half of these have IFs available for the recent years. *BMC Bioinformatics *was assigned its first IF for 2004. At 5.4, it places the journal second in the field, only marginally below the traditional competitor *Bioinformatics *(IF = 5.7), which has a 20-years' publishing history and is connected to a major learned society within this field of research (International Society for Computational Biology).

PLoS (Public Library of Science) is another example of a successful "open access" publishing strategy. It started publishing two open access journals in biology and medical research in 2003 and 2004 respectively [24]. PLoS Biology was assigned its first IF of 13.9 for 2004. In the ISI subject category "biology", it is thus placed at the number 1 position of 64 in its first year of reporting an IF. FASEB journal at position 2 has an IF of 6.8, but has been in circulation since 1987. Similarly, in the other SCI subject category ("biochemistry and molecular biology")in which PLOS Biology is listed, it ranks at position 8 out of 261. Monitoring the development of such journals' IF will inform the determination of the online-availability bias in the future. This effect will increase in the future with the availability of new search engines with deep penetration such as Google Scholar [25,26], allowing researchers to find relevant articles in an instant, and then choose those with immediately and freely available content over those with barriers, economic and otherwise.

### Accuracy of data capture by ISI

Investigations by *Nature *suggested a significant undercount of "citable" items in *Nature Genetics *in 1996 and an erroneous inclusion of "citable" items other than those defined by ISI itself for *Nature *in 2000 [4]. A more recent issue is undercounted citations to articles authored by consortia, rather than by a list of individual authors [27]. The article reporting the draft human genome sequence from the International Human Genome Sequence Consortium [28] is considered as a landmark paper published in *Nature *in 2001, but was surprisingly absent from the list of "hot papers" in biology, which are published regularly by ISI Science Watch [29]. The examination of the ISI's data showed that the ISI only considered citations to the full list of authors, led by Eric Lander of the Whitehead Institute for Biomedical Research at Cambridge, Massachusetts, and hence led to the grossly undercounted representation. The same applied to other prominent papers authored by consortia.

The accuracy of how citations are collected at ISI significantly influences the final published IF statistics. At ISI, data capture of journal papers is completed by optical character recognition software, important fields are highlighted manually, and the final tagging of every individual article is computerized. Algorithms have been designed to count valid citations. The simple involvement of two highlighted fields, such as the journal title and the year, makes the citation counting for the IF calculation easier. However, this raises systematic bias as citations cannot be matched to individual articles in real time. At this point an incorrect citation leaves its mark. For the field of environmental and occupational medicine, a recent study reported a prevalence of 3.35% incorrect citations; the respective articles receive an incorrect cite count, thus potentially reducing their journals' IF [30].

### IF is calculated for a whole journal whereas citations are to individual articles

The IF would reflect a journal's interest to the research community if citations were indeed distributed equally over all articles in the journal. However, this is not the case. Only a small percentage of articles are highly cited. Based on the analysis of three biochemical journals, Seglen [13] found that the most cited 15% of articles account for 50% of the citations and the most cited 50% of articles account for almost all citations (90%). These numbers were confirmed by a later study based on two cardiovascular journals [31]. The most recent study on articles published in *Nature *showed a similar high skew of citations: 89% of 2004's citations were generated by just 25% of *Nature*'s papers [32]. Apparently, researchers cannot solely depend on the IF to judge the quality of the journal.

Highly cited articles are found mostly in a small subset of journals, regardless of how parameters of the algorithm (e.g. average time-frame) are changed. In Garfield's view, these two combined effects strengthen the ISI's position as a means to point authors and readers to journals with true scientific impact [8]. The argument is that this effect justifies the fact that JCI is not all-inclusive, but rather selective. According to Garfield, JCI could still be considered comprehensive if it covered only the 500 most cited journals.

Invalid articles may pose a considerable bias on the journal IF. Retracted articles may continue to be cited by others as valid work. Pfeifer and Snodgrass [33] identified 82 completely retracted articles, analyzed their subsequent use in the scientific literature, and found that these retractions were still cited hundreds of times to support scientific concepts. Kochan and Budd [34] showed that retracted papers by John Darsee based on fabricated data were still positively cited in the cardiology literature although years had passed since retraction. Budd et al. [35] obtained all retractions from MEDLINE between 1966 and August 1997 and found that many papers still cited retracted papers as valid research long after the retraction notice.

Interesting papers, based on fraudulent data, may attract the scientific community's attention and be cited frequently, thus distorting the true impact of the journal that featured the sensational article. In a notable 2002 case of scientific fraud, Jan Hendrik Schön, a former researcher at Bell Laboratory, published "remarkable" findings on superconductivity, molecular electronics, and molecular crystals in several scientific journals, including *Science*, *Nature *and *Applied Physics Letters*. He was later found out to have falsified or fabricated data in 16 of 24 alleged cases of misconduct [36]. The data of 25 publications were implicated in the perpetuation of dubious claims. The findings of the investigation dismissed research results from "high impact" papers that had been promoted as major breakthroughs in the field.

### Active manipulation of IF

Owing to the preference authors and researchers give to high IF journals, editors may be tempted to artificially raise a journal IF. One very crude way to do so is by requesting author self-citation. In 1997, the journal *Leukemia *was accused of trying to manipulate its IF [37]. This first accusation came from Terry Hamblin, editor of *Leukemia Research*, a competitor to *Leukemia*. The evidence he was holding showed that *Leukemia *had asked authors who had submitted a paper to the journal to cite more articles from *Leukemia*. Later in 2002, Neuberger and Counsell [38] reported another similar case: they described how one journal editor suggested the inclusion of more references to that journal. In 2004, Sevinc [39] reported yet another incident. The influence of authors' choice of references distorts the perception of the journal within the scholarly community and is considered as highly unethical behavior.

## Alternative journal impact measures

The wide use of the IF, combined with obvious flaws, has motivated researchers in scientometrics to try to improve the algorithm for the calculation of the IF or to develop alternative journal citation measures altogether.

Van Leeuwen and Moed [20] have critically analyzed the use and validity of the ISI IF. They focused on four aspects: "non-citable" items included in the numerator of the IF calculation; the relative distribution of research articles; technical notes and reviews, different citing behavior across subject fields; and the fixed two-year citation window. They developed an alternative journal impact measure, the *Journal to Field Impact Score *(JFIS), to provide solutions to biases incurred from these four aspects. The JFIS includes research articles, technical notes, letters and reviews both in the numerator and the denominator. The JFIS also is field-normalized by comparing the journal's impact with the citation average in the fields it covers. The JFIS takes into account the relative distribution among the four types of distribution. Finally, the JFIS is computed based on a flexible and variable citation and publication window, and the selected publication window can in principle be set to any length. Despite the improvements that the JFIS has over the IF, van Leeuwen and Moed still suggested that more than one indicator should be used in bibliometric journal impact measurements.

Other researchers have focused on refining the ISI IF's limitations, such as the fixed two-year chronologic window. Asai [40] found that more accurate statistics could be calculated if the period count is based on months rather than a year. Accordingly, he proposed an *Adjusted Impact Factor *to count a weighted sum of citations per month over a time period of four years. Glänzel and Schoepflin [41] conducted a bibliometric study to analyze the time behavior of citations to articles published in seven journals in different subject fields including social sciences, chemistry, medicine and mathematics. The results suggested a three-year citation window to be a good compromise between fast growing disciplines and slowly aging theories.

Sombatsompop et al. [42] introduced the cited half-life into the IF calculation as an alternative to setting the citation window at an absolute number. The proposed indicator, the *Cited Half-Life Impact Factor *(CHAL-IF), is calculated by replacing the two-year citation window with the journal's cited half-life in the IF computation formula. This study was based on 34 journals in the Polymer Science Category from the ISI subject heading categories. The journal ranking based on the CHAL-IF was different from that based on the ISI IF. The average IF by the CHAL method achieved a better stability than that calculated by the standard ISI method. Rousseau [43] renamed the CHAL-IF to *Median Impact Factor *(MIF). He further generalized the MIF to create a *Percentile IF *(pIF). The MIF is a special case of the pIF with p set at 50%. These modified IFs are not meant to replace the ISI IF, but should rather be understood as a complementary assessment tool.

When ranking a list of journals within a subject discipline, it is inadequate to only compare the IF without consideration of subject bias. Hirst [44] introduced what he called the *Disciplinary Impact Factor *(DIF) to overcome this subject bias. It is based on the average number of times a journal was cited in a sub-field rather than the entire SCI database. A similar approach was chosen by Pudovkin and Garfield [45], who suggested a rank normalized impact factor to be calculated within each subject category. For any journal j, its rnIF is designated as rnIF(j) and equals (K - R-j + 1)/K, where R-j is the descending rank of journal j in its JCR category and K is the number of journals in the category. Ramírez et al. [46] proposed a renormalized IF which was calculated based on the maximum IF and median IF of each category. This quantitative parameter allows the direct comparison among different research areas without introducing other considerations. Sombatsompop [47,48] introduced a new mathematical index, the "Impact Factor Point Average" with the specific aim to allow across-field comparison of IF.

The above-mentioned variants of the IF may improve journal citation methodological aspects. As of now, no database makes use of these derivative algorithms. They are neither widely known nor accessible to the scientific community. There are some commercial alternative databases available that claim to overcome the intrinsic flaws of the SCI database.

The Euro-Factor (EF) database is a moderately successful example of citation analysis innovation. Targeting the language bias and perceived *USA-centricity *of the SCI database, the Euro-Factor™ (EF) [49] was proposed as an alternative to the ISI IF to meet the citation measurement demand of the European scientific community. The publishing company VICER [50] created the "Euro-Factor" database, in order to collect bibliometric data from biomedical journals in European countries. More than 500 journals were included by means of a peer-reviewed quality selection process. A new algorithm was designed to analyze the biometric relationship between European journals:



Unfortunately, VICER does not provide detailed explanation of the algorithm outside of the simple formula, which arbitrarily sets the EF-Coefficient at a value of 10. The formula does not further the understanding of how a Europe-specific ranking is achieved. The EFs of all European journals covered are calculated every year, and the list of EFs is available from VICER every January. According to VICER, the EF for *Lancet *and *Nature *in 2002 is 106.1 and 55 [49], whereas the ISI gives them IF of 15.4 and 30.4 respectively. In these two prominent examples, it seems somewhat naïve to speak of European journals, as both have editorial offices in the United States.

The Prestige Factor (PF) database possessed a dubious and short-lived existence. In an effort to challenge the ISI IF, in 2001 the "*Prestige Factor*" (PF) was launched at "PrestigeFactor.com". The PF was heralded as a superior assessment tool. It promised to measure the true value of academic journals by including original articles only and hosting a "superior" database compared to SCI. With only minor differences, such as the inclusion of original articles only and a three year citation count window, the underlying premise of both the IF and PF was identical [51]. One detailed analysis of the PF's social sciences subset found essential misrepresentations and misleading data on the company's website [52]. Concerns about the source of citations in the PF database were raised and led to doubts and competitive accusations. In 2002, the company was forced out of business in the wake of a threat from ISI to sue for intellectual property infringements.

## What question does the impact factor answer?

Strictly speaking, the journal IF only measures the average citation rate of all the "citable" articles (research articles, technical notes and reviews) in a journal. As such, IF is not a perfect tool to measure the journal quality. However, in most cases, it performs what it promises when various flaws are taken into active consideration. Ready accessibility and regular updates of the ISI IF provides the best available indicator for journal quality, accepted widely within the scientific community. Journals with the highest IF in each discipline are usually the most prestigious ones [8]. It can be considered as a general guideline that helps librarians determine which journals to purchase, helps authors to decide which journal to submit their work to, helps editors and publishers to assess their journals, and helps the funding agencies to shortlist applicants. Garfield [11] points out the IF's surrogate function as a measure of potential future impact of very recent publications, and as a safeguard against hiding ineffective research where funding may have been obtained through political connections rather than research quality. In Garfield's words: "impact simply reflects the ability of journals and editors to attract the best papers available" [53].

## What questions does the impact factor not answer?

The IF cannot assess the quality of individual articles, due to the qualitative variety of citations distributed in a journal [13,31,32]. A small proportion of articles count for a large percentage of citations. This means that a typical article in a high IF journal may not be cited more frequently than an average article in a relative low IF journal. As a result, IF alone is not able to judge the individual article's or author's performance.

Even under the assumption that citations were equally distributed among all articles, the IF would only measure the *interests *of other researchers in an article, but not the article's *importance *and *usefulness*. The *Guide to Clinical Preventive Services *by the US Preventive Services Task Forces (USPSTF) [54] is generally thought to be an example of top-level scientific evidence, the best available knowledge source. Nakayama et al. [55] showed that articles from "low impact factor" journals were also cited frequently in this guide, demonstrating the usefulness of those articles in providing clinical evidence.

In order to determine the relationship between citation factors and a trial's methodological quality, Berghmans et al. [56] analyzed citation indexes including the IF by assessing 181 eligible articles included in nine systematic reviews performed by the European Lung Cancer Working Party (ELCWP). The results showed that journals with higher citation factors did not publish higher quality clinical trials. Furthermore, several studies showed invalid articles continue to be cited after their retraction [33-35]. The high number of citations to these articles may raise the IF of the respective journals, yet this high IF cannot guarantee the significance and performance of an article published in such periodicals.

## How may different professional groups take into account the limitations of the impact factor?

Different professional groups need to take into consideration the inherent limitations of the IF. Librarians can use the IF to identify multidisciplinary journals, as a higher IF hints at wider acceptance of the journal. New and very specialized journals, however, must be assessed separately, as an IF might not yet be available or not reflect the importance of the journal within a given field of high specialization. The easiest way to assess the relative position of a particular journal within its field is to browse through the SCI's Subject Category and sort all journals by their IFs in a particular category. It should be noted that some journals may be classified as members of more than one category, and ranked differently across categories. When assessing new journals or journals from highly specialized disciplines, librarians should actively look for guidance from researchers at their institution that might be involved in that particular field of science.

Scientists hope to publish in "prestigious" and widely-read journals primarily to communicate their findings and achieve visibility with peers, enhancing their career prospects. While considering a journal submission target, the most important factors influencing authors' decision are the perceived reputation of the journal (often equated with the IF), closely followed by the international reach and inclusion in abstracting and indexing services [57-59]. As an indirect measure of these qualities, the IF has a place in the process of decision making [11], but should not be paramount. Thought should be given to how well the manuscript's topic fits the journal, the actual circulation numbers, and potential readership. As readers, scientists may customize IF analysis by including only citations from individually chosen trusted journals to other journals in the field and thus identify relatively unknown journals of interest to this research topic. This technique was suggested (and used) by Garfield in 1972 [60], but may be somewhat obsolete in an era when digital library readers can quickly access and scan the abstracts of interesting articles.

Editors and publishers must have a strong determination to publish valid articles without regard to the possibility of a potentially high citation count. Publishers must also analyze *how *articles are cited (Do citing authors agree or disagree? Do they cite a technique, or a conclusion? Are citations to valid articles, or was a retraction overlooked?) if they want to improve the quality of the research they publish. Editors might put additional effort in identifying the "best" quality papers, rewarding the successful author(s) a distinction of merit, useful when preparing an academic promotion portfolio [51]. BMC has recently introduced such a feature by labeling articles as "Highly accessed" if they are accessed more frequently than would be expected in the subject category. BMC does not, however, disclose the exact usage benchmark. In the end, nothing replaces innovative, and even good controversial research [11] as a promotional too for a scientific journal.

ISI cautions against the use of IF for the evaluation of individual researchers [4], yet funding agencies continue to track the IF record of applicants as an individual's investigator's assessment. Finland demonstrates an extreme example of IF canonized into law. Finnish government funding for university hospitals there partially depends on "publication points", which are derived from the IF of journals wherein the researchers publish their work [4]. Due to the IF's inability to compare individual articles, funding agencies should develop a detailed assessment of how an individual's work impacts on the scientific community, including how a submission decision was made. The IF is an indirect, affiliated measure of a researcher's work at best. A recent publication in a high-impact journal with high editorial standards and strict peer-review leads to the assumption of quality for the individual article [11]. Individual researcher assessments by funding agencies or potential employers would be best advised to make use of subject category-specific derivates of the IF, such as the rank-normalized IF [45].

## Conclusion

The present narrative review gives an introduction to the scientometrics of the ISI IF to non-specialist librarians, researchers and administrators. It describes the IF as a bibliometric tool with limited explanatory power. The IF must be used with full knowledge of its limitations and can then serve an indirect affiliated indicator of research quality. More precise information can be gained if some of the described alternative measures are appropriately used.

## Competing interests

The author(s) declare that they have no competing interests.

## Authors' contributions

Author 1 (PD) and author 3 (AM) participated in literature review, while author 1 (PD), author 2 (ML) and author 3 (AM) drafted the manuscript.
